# Proactive and retroactive interference with associative memory consolidation in the snail *Lymnaea* is time and circuit dependent

**DOI:** 10.1038/s42003-019-0470-y

**Published:** 2019-06-26

**Authors:** Michael Crossley, Frederick D. Lorenzetti, Souvik Naskar, Michael O’Shea, György Kemenes, Paul R. Benjamin, Ildikó Kemenes

**Affiliations:** Sussex Neuroscience, School of Life Sciences, University of Sussex, Brighton, BN1 9QG UK

**Keywords:** Learning and memory, Cellular neuroscience

## Abstract

Interference-based forgetting occurs when new information acquired either before or after a learning event attenuates memory expression (proactive and retroactive interference, respectively). Multiple learning events often occur in rapid succession, leading to competition between consolidating memories. However, it is unknown what factors determine which memory is remembered or forgotten. Here, we challenge the snail, *Lymnaea*, to acquire two consecutive similar or different memories and identify learning-induced changes in neurons of its well-characterized motor circuits. We show that when new learning takes place during a stable period of the original memory, proactive interference only occurs if the two consolidating memories engage the same circuit mechanisms. If different circuits are used, both memories survive. However, any new learning during a labile period of consolidation promotes retroactive interference and the acquisition of the new memory. Therefore, the effect of interference depends both on the timing of new learning and the underlying neuronal mechanisms.

## Abstract (web summary)

Michael Crossley et al. use the snail *Lymnaea* to investigate the factors involved in determining which memories are remembered or forgotten when multiple learning events happen close together. They find that timing of events and whether the same neuronal mechanisms are used are key factors in determining whether interference will occur.

## Introduction

It is well documented that during memory consolidation, the time-dependent stabilization of newly acquired memory, the memory trace is vulnerable to disruption by a variety of amnestic influences^1–5^. One such disruptive influence is retroactive interference, where the acquisition of a new memory during consolidation leads to the forgetting of the original one^6,7^. However, new learning during memory consolidation can also result in the inability to consolidate the new information into stable memory (proactive interference), e.g., when an original memory is retained at the expense of a new one. Despite the large body of literature on both retroactive and proactive interference, the factors that determine which, if either, of these two different types of interference will affect memory consolidation and how they will affect it are poorly understood. For example, it is not clear if interference is required to erase the memory trace so that it can no longer be retrieved or it is only the expression of the memory that has been suppressed by retroactive or proactive interference.

Here we tested the hypothesis that the direction of interference (proactive or retroactive) between a memory trace and new learning during its consolidation is dependent upon the timing of the interference in the consolidation sequence of the original memory. We also examined whether the original and the new memory are encoded by plastic changes in the same or different circuits and whether it depended on the type of learning used for interference. To address these questions, we used the pond snail, *Lymnaea stagnalis*, which can learn simple associations after a single pairing of a conditioned and unconditioned stimulus, leading to long-term memory that lasts up to 19 days^8,9^. Furthermore, their neurons are large and re-identifiable, and the underlying circuitry of the conditioned behaviors studied here have been extensively characterized^10–14^, aiding in the identification of learning-induced changes in neural activity responsible for encoding memories^9,15–18^. In *Lymnaea*^19^ and other systems^20–22^, the strength of memory fluctuates during the process of consolidation, resulting in temporary lapses in memory expression when trained animals are less responsive to the conditioned stimulus and more prone to forgetting. In *Lymnaea*, lapses in memory coincide with phase-transitions in the early stages of memory consolidation^19^. This allowed us to compare the effect of interference when new learning occurs during either a labile phase-transition (lapse) or no phase-transition (non-lapse) period within the same consolidation sequence.

In this study we found that whether long-term memory (24 h) for the original appetitive association was preserved after new appetitive learning depended on the timing of the second training. New appetitive learning during a lapse point 2 h post-training, a phase-transition between early and late intermediate-term memory^19^, retroactively interfered with the original memory leading to the emergence of a new memory. By contrast, after appetitive learning at a non-lapse point 1 h post-training, the first-acquired memory survived while the second memory was absent (proactive interference). When a different type of learning, aversive conditioning, took place in the lapse period of the appetitive memory, it again retroactively interfered with the original memory and the new aversive memory was acquired. However, when the aversive memory was induced during the non-lapse period, both memories survived, so there was no proactive interference. We characterized neuronal changes induced by the appetitive and aversive learning and demonstrate that they occur in distinct circuits, permitting the concurrent consolidation of both memories. The overlapping consolidation of two appetitive memories in the same memory circuit, however, only allows the formation of a single memory due to competitive interactions between the two related memories. Our study therefore reveals that the type of interference depends on the timing of the new learning as well as the underlying neural circuits by which the memories are encoded.

## Results

### How memories interfere depends on when new learning occurs

To test the hypothesis that the timing of the second training is a key factor in determining whether proactive or retroactive interference occurs, we employed a paradigm that was designed to investigate competition between two different cues that are trained apart, but have a common outcome, e.g., the same type of conditioned response^23^. Specifically, we performed a dual appetitive classical conditioning procedure involving two neutral chemical stimuli, gamma-nonalactone or amyl acetate (the conditioned stimuli), both of which were paired with a salient food stimulus (sucrose, the unconditioned stimulus) that activates the well-identified feeding circuit of *Lymnaea*^11,24,25^. A single pairing of either conditioned stimulus with the unconditioned stimulus leads to the formation of long-term appetitive memory, notably, with non-lapse and lapse periods observed at the same time points during memory consolidation^8,19^ (Supplementary Fig. 1). Consecutive training with these two appetitive paradigms was employed to establish the type of interference occurring during a non-lapse versus a lapse period of the same consolidation sequence.

**Fig. 1:**
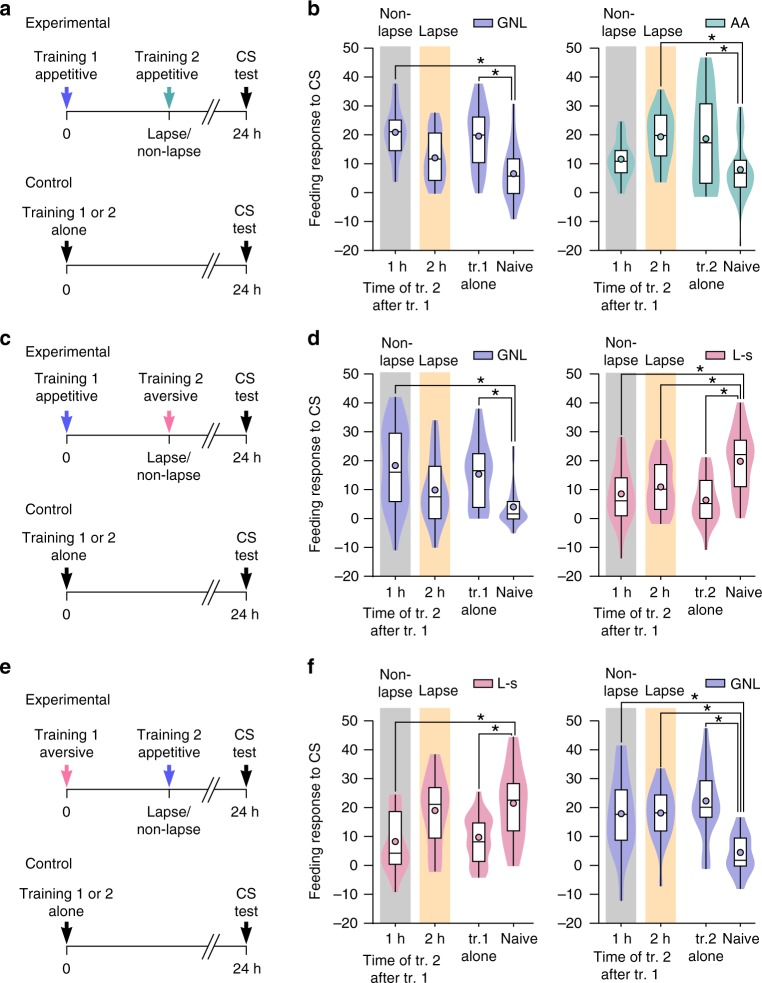
Proactive and retroactive interference and parallel consolidation of associative memories after single-trial conditioning. **a** Simplified time-line of dual appetitive conditioning paradigm. **b** Animals showed a greater response to the first conditioned stimulus (CS), gamma-nonalactone (GNL), after first training alone (*n* = 40) or when second training occurred during the non-lapse (1 h: *n* = 32) but not the lapse (2 h: *n* = 29) compared to naïve controls (*n* = 35). Responses to the second conditioned stimulus, amyl acetate (AA), were greater in animals that received second training alone (*n* = 24) or second training during the lapse (2 h: *n* = 31) but not the non-lapse (1 h: *n* = 32) compared with naïve controls (*n* = 40). Violin plots show density of data extending from minimum to maximum values. Internal boxplots show median and interquartile range (first and third quartile). Whiskers represent minimum to maximum values. Circles show the mean. **c** Simplified time-line of appetitive followed by aversive training. **d** Animals showed a greater response to the appetitive conditioned stimulus (gamma-nonalactone) after appetitive training alone (*n* = 30) or when aversive training occurred during the non-lapse (1 h: *n* = 29) but not the lapse (2 h: *n* = 30) compared with naïve controls (*n* = 28). Responses to the aversive conditioned stimulus, L-serine (L-s), were lower in animals that received aversive training during the lapse (2 h: *n* = 29), non-lapse (*n* = 30) and after aversive training alone (*n* = 30) compared with naïve controls (*n* = 29). **e** Simplified time-line of aversive followed by appetitive training. **f** Animals showed a lower response to the aversive conditioned stimulus after aversive training alone (*n* = 20) or when appetitive training occurred during the non-lapse (1 h: *n* = 20) but not the lapse (2 h: *n* = 20) compared with naïve controls (*n* = 20). Responses to the appetitive conditioned stimulus were greater in animals that received appetitive training during the lapse (2 h: *n* = 20), non-lapse (*n* = 16) and after appetitive training alone (*n* = 20) compared with naïve controls (*n* = 20)

Animals were trained with gamma-nonalactone + sucrose (referred to as ‘first appetitive training’), and then a second training of amyl acetate + sucrose (referred to as ‘second appetitive training’) was applied at either a non-lapse (1 h) or lapse (2 h) point of the first memory. They were then tested for the presence of long-term memory 24 h after the first training (Fig. 1a). Animals that received the second appetitive training at the non-lapse point retained a memory for the first conditioned stimulus (gamma-nonalactone) but not for the second conditioned stimulus (amyl acetate) (Fig. 1b, ‘non-lapse’), indicative of proactive interference, whereas animals that received the second appetitive training during the lapse point had a memory for the second conditioned stimulus, but not the first conditioned stimulus (Fig. 1b, ‘lapse’), indicative of retroactive interference (gamma-nonalactone tested animals: One-way ANOVA, *p* < 0.001 (*F*_(3,132)_ = 17.27), Bonferroni test: 1 h vs naïve *p* < 0.001, first training alone vs naïve *p* < 0.001, 2 h vs naïve *p* > 0.05. Amyl acetate tested animals: One-way ANOVA, *p* < 0.001 (*F*_(3,123)_ = 9.72), Bonferroni test: 2 h vs naïve *p* < 0.001, second training alone vs naïve *p* < 0.001, 1 h vs naïve *p* > 0.05).

It was possible that the absence of memory after interference was due to the animal’s inability to simultaneously store two similar long-term memories. To examine this, we trained animals with both types of appetitive paradigms but spaced 24 h apart, allowing the first memory to fully consolidate prior to the second training (Supplementary Fig. 2a). Each animal was tested for its response to both conditioned stimuli 24 h after the second training. To ensure that the order of testing did not affect the animal’s response, one group was tested for their response to gamma-nonalactone first and then amyl acetate 1 h later, whilst a second group received them in the reverse order. Both groups showed a greater response to both conditioned stimuli compared with naïve animals indicating the presence of two memories in the same animal (Supplementary Fig. 2b).

An alternative hypothesis is that the inability to consolidate both memories concurrently is due to competition between two similar memories that utilize the same underlying neural circuit. We investigated whether the same rules of interference applied when the second training employed a paradigm that utilizes a circuit different from the one activated by the first learning. Aversive conditioning of feeding in *Lymnaea* is processed by a neuronal circuit not involved in food-reward conditioning^26^; therefore an aversive paradigm was used to test the competition hypothesis. A single pairing of L-serine (an appetitive stimulus, see Fig. 1d) with quinine (an aversive stimulus that inhibits feeding^27^) induced long-term memory, expressed as a decreased feeding response to the conditioned stimulus, when tested at 24 h compared with naïve controls (naïve feeding difference score: 21.1 ± 2.5, *n* = 16, L-serine + quinine feeding difference score: 9.8 ± 2.1, *n* = 17, unpaired *t*-test, *p* = 0.0016, *t* = 3.47, df = 31). Notably, during the consolidation of the aversive memory, lapses occurred at the same time points as during appetitive memory formation, demonstrating that lapses are a general feature during consolidation in *Lymnaea* (Supplementary Fig. 3). Next, animals were trained with gamma-nonalactone + sucrose (appetitive training), followed by aversive training at the same non-lapse or lapse points of the first memory as in the dual appetitive paradigm (Fig. 1c). Aversive training during the non-lapse point gave rise to both an appetitive and aversive memory (Fig. 1d, ‘non-lapse’), indicating the absence of proactive interference, whereas aversive conditioning during the appetitive memory lapse resulted in an aversive memory, but not an appetitive memory (Fig. 1d, ‘lapse’) (gamma-nonalactone tested animals: One-way ANOVA, *p* < 0.001 (*F*_(3,113)_ = 9.47), Bonferroni test: 1 h vs naïve *p* < 0.001, appetitive alone vs naïve *p* < 0.001, 2 h vs naïve *p* > 0.05. L-serine tested animals: One-way ANOVA, *p* < 0.001 (*F*_(3,114)_ = 12.13), Bonferroni test: aversive alone vs naïve *p* < 0.001, 1 h vs naïve *p* < 0.001, 2 h vs naïve *p* < 0.01).

To test whether the lack of proactive interference between the appetitive and the aversive memory was due to the latter being stronger than the former, and therefore less prone to interference, we reversed the order of training, performing aversive followed by appetitive training (Fig. 1e). With this reversed paradigm, we observed the same pattern of memory interference as when the appetitive training preceded the aversive training (L-serine tested animals: One-way ANOVA, *p* < 0.001 (*F*_(3,76)_ = 7.34), Bonferroni test: 1 h vs naïve *p* < 0.001, aversive alone vs naïve p < 0.01, 2 h vs naïve *p* > 0.05. Gamma-nonalactone tested animals: One-way ANOVA, *p* < 0.001 (*F*_(3,72)_ = 10.18), Bonferroni test: appetitive alone vs naïve *p* < 0.001, 1 h vs naïve *p* < 0.01, 2 h vs naïve *p* < 0.001) (Fig. 1f). Taken together, these results demonstrate that the induction of a new associative memory during the lapse of the first memory causes retroactive interference regardless of whether the second training paradigm is appetitive or aversive. However, during the non-lapse period proactive interference only occurs when the second training paradigm is similar to the first. With a dissimilar paradigm, dual memory consolidation occurs. We next sought to identify possible neural mechanisms underlying these differences in the behavioral expression of one or the other type of memory depending on the paradigms used.

### How two memories interfere depends on the circuits they use

**Fig. 2:**
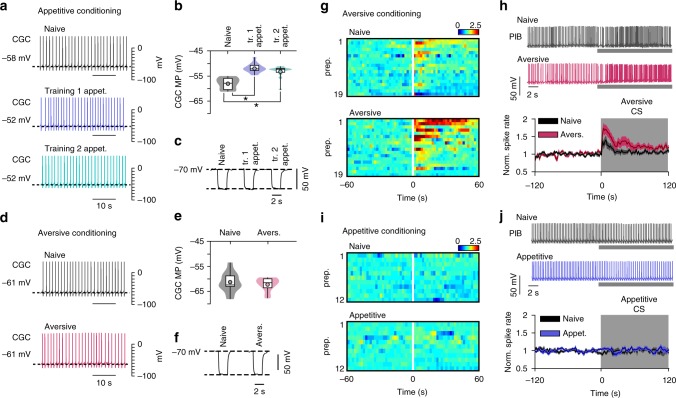
Different neuronal substrates for appetitive versus aversive learning. **a** Representative traces of CGC activity 24 h after animals received first or second appetitive training alone and from naïve animals. **b** CGCs from both first (*n* = 12) and second (*n* = 13) appetitive training groups were depolarization compared to naïve controls (*n* = 13) but not compared with each other. Violin plots show density of data extending from minimum to maximum values. Internal boxplots show median and interquartile range (first and third quartile). Whiskers represent minimum to maximum values. Circles show the mean. **c** CGC membrane resistance was not different between conditions. **d** Representative traces of CGC activity from aversively conditioned and naïve animals. **e** There was no significant difference in membrane potential between the two conditions (*n* = 12 for both). **f** Membrane resistance was not significantly different between conditions. **g** Heat plots of normalized PlB activity in response to aversive conditioned stimulus (CS) in preparations from aversively trained and naive animals. Data organized from high to low PlB activity after the conditioned stimulus. White line represents start of conditioned stimulus. **h** Representative traces and normalized line plot of PlB spike frequency in response to the aversive conditioned stimulus. Line and shading show mean ± standard error of the mean, respectively. **i** Heat plots of normalized PlB activity in response to appetitive conditioned stimulus in preparations from appetitive trained and naïve animals. **j** Representative traces and normalized line plot of PlB spike frequency in response to the appetitive conditioned stimulus. Abbreviations: appet, appetitive; avers, aversive; tr, training; MP, membrane potential; norm, normalized; prep, preparation

We hypothesized that the inability to simultaneously consolidate two appetitive memories is due to both being encoded within the same memory circuit, whereas the aversive association’s use of a distinct circuit mechanism permits dual consolidation outside the lapse periods. A previously identified cellular change involved in long-term memory after appetitive conditioning in *Lymnaea* is the persistent depolarization of a modulatory neuron in the feeding network, the CGCs (cerebral giant cells)^9,28,29^. The learning-induced depolarization gates-in the conditioned stimulus input to feeding command-like interneurons, which in trained animals results in the activation of the feeding network^9^. Here, we demonstrate that both types of appetitive training induce the same persistent depolarization compared with naïve controls (One-way ANOVA, *p* < 0.001 (*F*_(2,35)_ = 26.3), Bonferroni test: first training alone vs naïve *p* < 0.001, second training alone vs naïve *p* < 0.001, first training alone vs second training alone *p* > 0.05) (Fig. 2a–c). We next tested whether aversive conditioning affected the CGCs and found no significant change in their membrane potential compared with naïve controls (unpaired *t*-test, *p* = 0.53, *t* = 0.63, df = 22) (Fig. 2d–f). There was no change in CGC membrane resistance or spike characteristics after either appetitive or aversive conditioning (CGC membrane resistance: appetitive training, One-way ANOVA, *p* = 0.17 (*F*_(2,34)_ = 1.77). Aversive training, unpaired *t*-test, *p* = 0.67, *t* = 0.44, df = 22.) (Fig. 2c, f; Supplementary Fig. 4a, b). Therefore, both appetitive paradigms induce the same cellular change, whereas aversive training does not affect the properties of this neuron suggesting that the memory is encoded in another circuit.

We next sought to identify changes induced by aversive conditioning. Since the conditioned response was a reduction in feeding, we reasoned that it might be due to an enhanced inhibitory effect originating from the defensive-withdrawal circuit. One candidate neuron for this is the PlB interneuron^30^ that connects the withdrawal and feeding circuits and its activation by aversive stimuli is sufficient to inhibit feeding^31^. In isolated brain preparations from aversively conditioned animals, an in vitro analogue of the conditioned stimulus (see Methods) caused a significant increase in PlB firing rate compared with naïve controls (Mann Whitney test, *p* = 0.029, *U* = 106) (Fig. 2g, h), as well as a lower expression of fictive feeding cycles (an in vitro correlate of the conditioned response) (Supplementary Fig. 4c, d). PlB firing rates before the conditioned stimulus were not significantly different between conditions (unpaired *t*-test, *p* = 0.94, *t* = 0.08, df = 36). CGC responses to the conditioned stimulus showed no change after aversive conditioning (Supplementary Fig. 4e). We tested whether PlB activity was altered after appetitive conditioning but found no change in PlB firing rates in response to the appetitive conditioned stimulus (Mann Whitney test, *p* = 0.39, *U* = 56.5) or in its firing rates before the conditioned stimulus (unpaired *t*-test, *p* = 0.38, *t* = 0.89, df = 22) (Fig. 2i, j). However, preparations derived from appetitively conditioned animals still showed a greater fictive feeding response to gamma-nonalactone compared to naïve controls (Supplementary Fig. 4f, g). These results demonstrate that aversive learning causes an increase in an inhibitory pathway, distinct from the neural changes underpinning appetitive memories. Taken together, these results suggest that competition within the same memory circuit is a limiting factor in the animals’ ability to consolidate multiple similar memories. Such competition does not affect the consolidation of dissimilar memories that rely on different circuit mechanisms, accounting for the lack of proactive interference of the appetitive and aversive memories at the non-lapse point.

### Retroactive interference requires new learning in general

**Fig. 3:**
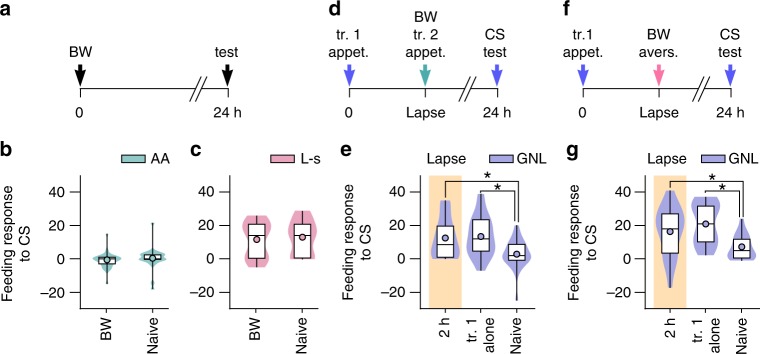
The acquisition of new memory is required for retroactive interference. **a** Time-line of backwards conditioning (BW) paradigm. **b** BW presentation of sucrose and amyl acetate (AA) does not elicit a significantly greater response to the conditioned stimulus (CS) compared with naïve controls when tested at 24 h (BW: *n* = 24, naïve: *n* = 21). Violin plots show density of data extending from minimum to maximum values. Internal boxplots show median and interquartile range (first and third quartile). Whiskers represent minimum to maximum values. Circles show the mean. **c** BW presentation of quinine and L-serine (L-s) does not induce an aversive memory (BW: *n* = 19, naïve: *n* = 20). **d** Time-line of BW with sucrose and amyl acetate during the lapse of the first appetitive memory (gamma-nonalactone (GNL) paired with sucrose). **e** BW during the lapse did not affect long-term memory compared with naïve controls (2 h: *n* = 22, first training alone: *n* = 21, naïve: *n* = 24). **f** Time-line of BW with quinine and L-serine during the lapse of the first appetitive memory (gamma-nonalactone paired with sucrose). **g** BW during the lapse did not affect long-term memory compared with naïve controls (2 h: *n* = 20, first training alone: *n* = 20, naïve: *n* = 20)

We next sought to dissect what aspect of the second training was responsible for retroactive interference at lapse points. We tested whether the induction of a second associative memory was necessary to block the first memory or whether simply the presentation of the conditioned and unconditioned stimuli during the second training was sufficient to act as a memory disruptor. To test this, we performed backwards presentation of the unconditioned stimulus + conditioned stimulus (referred to as BW) (Fig. 3a), which did not result in long-term memory using either the appetitive (Mann Whitney test, *p* = 0.07, *U* = 172.5) or aversive protocols (Mann Whitney test, *p* = 0.67, *U* = 174.5) (Fig. 3b, c), confirming that BW paradigms do not induce associative memory. Next, we performed either appetitive or aversive BW conditioning at a lapse point of the first memory and found that neither had an effect on the expression of the first memory (appetitive BW: Kruskal–Wallis test, *p* = 0.0043, *H* = 10.88; Dunn’s test, BW at 2 h vs naïve *p* < 0.05 and first training alone vs naïve *p* < 0.01. Aversive BW: Kruskal–Wallis test, *p* = 0.001, *H* = 13.81; Dunn’s test, BW at 2 h vs naïve *p* < 0.05, first training alone vs naïve *p* < 0.001) (Fig. 3d–g). These results suggest that the induction of a second associative memory is necessary for retroactive interference with the first memory.

**Fig. 4:**
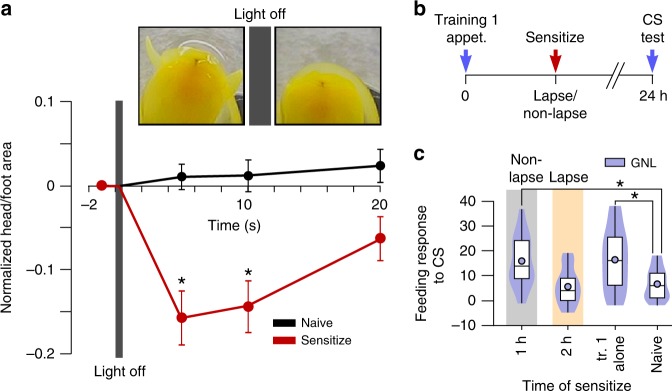
New non-associative learning is sufficient for retroactive interference. **a** Tactile stimulation of the head induces short-term sensitization. 10 min after tactile stimulation, a brief light-off stimulus was presented to the animal (inserted image) and their withdrawal response measured. Animals showed a significant increase in the withdrawal response to the light off stimulus, which lasted for 10 s after the stimulus (left image, before stimulus; right image, after stimulus) (*n* = 15). Naïve animals showed no significant withdrawal response to the same stimulus (*n* = 15). Data shows mean ± standard error of the mean. **b** Time line of application of sensitizing stimulation after appetitive conditioning. **c** Sensitization during the lapse, but not during the non-lapse, significantly disrupts the animal’s memory for the conditioned stimulus (CS), gamma-nonalactone (GNL), when tested at 24 h (first training alone: *n* = 29, 2 h: *n* = 19, 1 h: *n* = 22, naïve: *n* = 29). Violin plots show density of data extending from minimum to maximum values. Internal boxplots show median and interquartile range (first and third quartile). Whiskers represent minimum to maximum values. Circles show the mean

This raised the question whether it is specifically new associative learning or new learning in general that can cause retroactive interference. To address this we used a non-associative paradigm as the second training. We demonstrated that strong tactile stimulation of the head leads to a sensitized withdrawal response to a brief ‘light off’ stimulus (Fig. 4a). This brief stimulus did not trigger a withdrawal response in naïve animals (Repeated-measures ANOVA, *p* = 0.62 (*F*_(3,42)_ = 16.58)) (Fig. 4a). By contrast, animals that were exposed to strong tactile stimulation 10 min before the ‘light off’ stimulus showed a significant withdrawal response (Repeated-measures ANOVA, *p* < 0.001 (*F*(3,42) = 0.54), Dunnett’s test: before vs 5 s *p* < 0.001, before vs 10 s *p* < 0.001, before vs 20 s *p* > 0.05) (Fig. 4a). Thus, strong tactile stimulation of the head causes sensitization, a form of non-associative learning.

Next, we applied the sensitizing stimulation at the lapse point of the appetitive memory (Fig. 4b). We showed that it retroactively interfered with the associative memory, whereas when applied at the non-lapse point, the memory was unimpaired (One-way ANOVA, *p* < 0.001 (*F*(3,78) = 7.191), Bonferroni test: first training alone vs naïve *p* < 0.001, 1 h vs naïve *p* < 0.01, 2 h vs naïve *p* > 0.05) (Fig. 4c). Therefore, acquisition of either an associative or non-associative memory during the lapse period retroactively interferes with the original associative memory.

### Retroactive interference disrupts memory consolidation

**Fig. 5:**
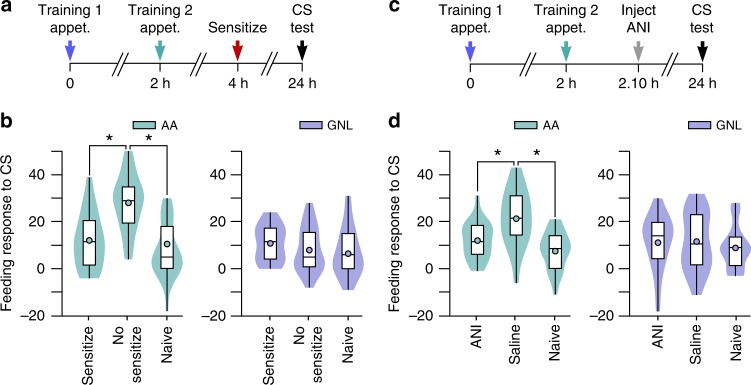
Blocking the second memory does not lead to the recovery of the first memory. **a** Time-line of experiment. Animals received the first training followed by the second training during the 2 h lapse. Sensitizing stimulation were applied 2 h after the second training (4 h after the first training). Animals were then tested for their response to either conditioned stimulus (CS), gamma-nonalactone (GNL) or amyl acetate (AA), at 24 h. **b** Sensitization was sufficient to block the second memory, whereas no sensitizing stimuli resulted in a strong second memory compared with naïve controls (sensitization: *n* = 22, no sensitization after second training: n = 24, naïve: *n* = 30). There was no increased response to gamma-nonalactone beyond naïve levels at 24 h despite the blocking of the second memory. Animals that received the second training without sensitization also did not have a significantly different response to gamma-nonalactone compared to naïve controls (sensitization: *n* = 22, no sensitization after second training: *n* = 18, naïve: *n* = 27). Violin plots show density of data extending from minimum to maximum values. Internal boxplots show median and interquartile range (first and third quartile). Whiskers represent minimum to maximum values. Circles show the mean. **c** Time-line of injection of anisomycin (ANI) after the second appetitive training. **d** ANI blocked the consolidation of the second memory, whereas saline injection resulted in a strong second memory (ANI: *n* = 20, saline: *n* = 20, naïve: *n* = 22). ANI after the second training did not recover the first memory when tested at 24 h (ANI: *n* = 20, saline: *n* = 20, naïve: *n* = 20)

We next tested whether the apparent replacement of the first memory by the second was due to retroactive interference disrupting the consolidation of the original memory or due to the suppression of its expression by the second memory. If the first memory could not be recovered by blocking the second one, this would indicate that its consolidation was disrupted. However, if it could be recovered, this would indicate that the expression of the first memory trace is actively suppressed by the co-existing second memory. To test this, second appetitive training was performed 2 h after the first appetitive training to interfere with the first memory. Sensitizing stimulation was then applied 2 h later, at a lapse in the consolidation of the second learning (Fig. 5a) to block the second memory (Fig. 5b). This ensured that the sensitizing stimulation occurred at a lapse point of the second memory (2 h) but a non-lapse point of the first memory (4 h) (Supplementary Fig. 5a, b shows that the sensitizing stimulation is sufficient to block the second appetitive memory). Application of the sensitizing stimulation alone 4 h after the first appetitive training has no effect on the first memory (Supplementary Fig. 5c, d). Although this paradigm was successful at blocking the second memory, expression of the first memory was not restored at 24 h (Fig. 5b, gamma-nonalactone). By contrast, when no sensitizing stimulation was applied, there was the expected disruption of the first memory and acquisition of the second one (amyl acetate tested animals: One-way ANOVA, *p* < 0.001 (*F*_(2,73)_ = 21.11), Bonferroni test: no sensitization vs naïve *p* < 0.001, no sensitization vs sensitization *p* < 0.001, sensitization vs naïve *p* > 0.05. Gamma-nonalactone tested animals: One-way ANOVA, *p* = 0.33 (*F*_(2,64)_ = 1.12)) (Fig. 5b).

The use of the sensitization protocol allowed us to conclude that when the second memory was blocked at its 2 h lapse point, the first memory did not re-emerge. However, since the blocking of the second memory with sensitization could only be successfully performed at the memory lapse 2 h after the second training and we only knew that it erased the second memory when tested at 24 h, we needed another method that quickly blocks memory formation and leads to the erasure of the second memory at an early stage. Such a method would establish whether the blocking of only the earliest processes of the consolidation of the second memory would rescue the first memory. We therefore utilized pharmacological methods to block the early consolidation of the second memory. Treatment with the translational inhibitor anisomycin (ANI) rapidly blocks the synthesis of new proteins in the *Lymnaea* brain^32^ and its post-training application prevents the expression of memory from as early as 1 h after conditioning^19^ as well as its further consolidation into long-term memory^32^. Animals were injected with ANI or saline 10 min after the second appetitive training (2 h 10 min after first appetitive training) and tested for long-term memory (Fig. 5c). ANI injection alone at 2 h 10 min had no effect on the expression of the first memory (Supplementary Fig. 5e, f) but it had successfully blocked the second memory (Fig. 5d). However, this early intervention failed to rescue the first memory (Fig. 5d), indicating that it was indeed disrupted by the second memory within an hour after the second training. Saline injected animals showed the expected memory disruption (amyl acetate tested animals: One-way ANOVA, *p* < 0.001 (*F*_(2,59)_ = 11.09), Bonferroni test: saline vs naïve *p* < 0.001, ANI vs saline *p* < 0.01, ANI vs naïve *p* > 0.05. Gamma-nonalactone tested animals: One-way ANOVA, *p* = 0.75 (*F*_(2,57)_ = 0.295)) (Fig. 5d). These experiments suggest that retroactive interference happens within an early time window after the acquisition of the second memory. We conclude that blocking the second memory does not lead to the expression of a ‘suppressed’ first memory trace. These experiments therefore support the conclusion that, at least at the behavioral level, the second memory effectively replaces the first memory.

## Discussion

Memory consolidation is a crucial but vulnerable phase of learning when interference can result in the erasure of newly acquired information (retroactive control) or previous learning can affect the success of the acquisition of a second memory (proactive control). This dual control framework has been suggested by Braver^33^, whereby retroactive interference can serve as a late correction mechanism while proactive interference can protect the original memory trace. There are several studies addressing questions concerning the existence and ecological advantages of these mechanisms at the cognitive level^6,34–37^ but their neurobiological underpinnings remained largely unknown. Here we investigated these processes at both the behavioral and neurophysiological level in the well-characterized nervous system of *Lymnaea*.

**Fig. 6:**
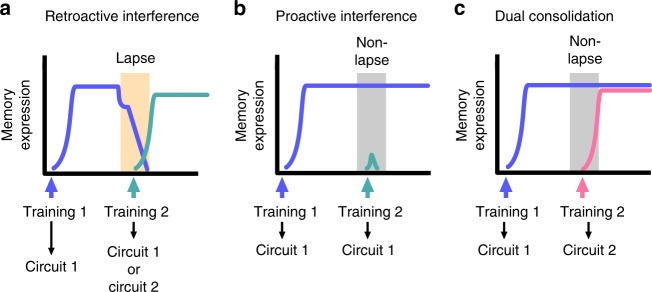
A simplified schematic of the interactions between memories during consolidation. **a** New learning (training 2) during a lapse point leads to the disruption of the first memory (retroactive interference) regardless of the whether the new memory is encoded within the same of a different circuit as the first memory. **b** When new learning occurs during a non-lapse point of the first memory and both memories are encoded in the same circuit, the first memory is retained and the second not acquired (proactive interference). **c** Acquisition of a second memory at a non-lapse point that activates a different circuit from the one triggered by the first learning does not lead to proactive interference and allows dual memory consolidation

A major advantage of this system is the brevity of the single-trial conditioning (2 min) that allowed us to both interfere with and test the memory trace at sharply timed intervals. Detailed knowledge of the memory phases and the timeline of fluctuations in memory strength during consolidation after associative training in *Lymnaea*^19^ provided us with the opportunity to test the effect of proactive or retroactive interference when the consolidating memory is either in a stable or labile stage. Our results revealed that whether proactive or retroactive control is activated depends on the timing of the second training (Fig. 6a, b). Induction of a second memory during a labile phase of consolidation of the first memory leads to diminished response to the first conditioned stimulus at 24 h (Fig. 6a), thus the second training activates retroactive control mechanisms. Furthermore, this lack of detectable long-term memory was not dependent on the nature of the second training, both associative (appetitive or aversive) and non-associative learning (sensitization) at the lapse point resulted in forgetting of the first learning while remembering the second. Similar efforts were taken to dissect the time-dependent effect of interference with working memory by using repetitive transcranial magnetic stimulation either at an early (0–250 ms) or a later (500–750 ms) phase of consolidation in humans^38^. Although the results suggested that interference at these phases had differential effects, the lack of precise time resolution prevented the establishment of solid conclusions. Several other human studies also suggest that proactive and retroactive control may involve potentially independent mechanisms with distinct temporal dynamics (for review see^33^) but the complexity of the human cognitive system and the difficulty of precise timing and resolution of electrophysiological measurements limit the scope of these experiments. Work on the European starling has also demonstrated that memories can be interfered with by learning similar experiences to the original memory, but it was found that both memories were impaired when tested that same day, indicating both proactive and retroactive interference^39^. Importantly, retroactive interference occurred immediately after the second training, whereas proactive interference was delayed^40^. Therefore, the proactive mechanism did not prevent the acquisition of the new memory, but rather interfered with its ongoing consolidation. By blocking the second memory at an early time point after retroactive interference in *Lymnaea* we too found that the first memory was disrupted at an early stage after the second training. Whether proactive interference impedes new learning or impairs its retention is yet to be determined in *Lymnaea*.

To further understand the effect of timing of interference on long-term memory formation, we also tested if new learning during a more stable phase of consolidation might result in the retention of the first memory. Indeed, when an appetitive memory interfered with another appetitive memory at a non-lapse point then the first memory was protected by a proactive control mechanism while the second memory could not be retrieved a day later (Fig. 6b). Interestingly, when the second training was applied at the same time point but was aversive rather than appetitive, then both memories survived (Fig. 6c). These results show that survival of a second consolidating associative memory depends not just on the timing but importantly also on the nature of the second learning. When the interference is due to another type of associative learning that is fundamentally different in nature from the first one, both memories are capable of consolidating in parallel. In *Neohelice* crabs, an appetitive and aversive paradigm utilized in a single training session induces separate appetitive and aversive memories that compete during retrieval but not during acquisition^36^. The results indicated that both memories consolidated in parallel, similar to our results using consecutive appetitive and aversive training during a non-lapse period. However, unlike in the crab^36^, in *Lymnaea* there was no competition during the retrieval of the appetitive and aversive memories. In human studies, a serial reaction time task utilizes a mix of procedural and declarative components. Although acquired in parallel, there is competition between the two memories during their consolidation, with the declarative one inhibiting the off-line processing of the procedural one^41^. A likely explanation for the differences seen here is that in *Lymnaea* there was a delay in applying the two dissimilar training paradigms, so that there was no conflicting information present during the acquisition phase of either type of memory.

There are several biological mechanisms and biochemical pathways suggested to be involved in interference-based active forgetting but without experimental evidence for the erasure of the memory engram^42^. Here we show that blocking the second memory does not rescue the first memory. Although this finding lends support to the notion that the first memory is replaced, not simply suppressed by the second one, we cannot rule out that although not expressed at the behavioral level, some physiological trace of the original memory survives in the circuit encoding it. In *Drosophila*, when an aversive olfactory memory is extinguished by re-exposing trained flies to the conditioned stimulus without the expected punishment, intracellular calcium traces for both the original aversive and the new extinction memory co-exist in different places in the mushroom body output network^43^. In *Aplysia*, memories that have been erased at both the behavioral and synaptic level can be reinstated by a training paradigm which is insufficient to induce memory on its own^44^, suggesting that the memory or at least a priming signal for new memory formation remains stored, likely due to epigenetic changes in the nuclei of the pre- and/or postsynaptic neurons of the memory circuit^45,46^. Further work will be needed to clarify whether similar cellular and molecular traces of the original memory are present in *Lymnaea* after erasure due to retroactive interference.

The next question we investigated was whether there are separate circuit and neural mechanisms mediating proactive and retroactive control. Our results indicate that when both the first and second learning activates the same circuit there is competition between the two consolidating memories. As described earlier^9^, a persistent non-synaptic change in the CGC is involved in long-term appetitive memory. Here we investigated whether this learning-induced change was linked to the retroactive and/or proactive control mechanism. Our results demonstrate that both appetitive paradigms induce in the same CGC depolarization. Since the long-term results of both trainings are the same, it suggests that when in competition, only one of the memories can win exclusively and processes induced by the losing memory trace are halted by the winner. The dominance of one or the other memory trace in this case, when the same circuitry is activated by both, only depends on the timing of the interference. When the first memory is weak at the time of the induction of the second one, then processes triggered by the first training leading to CGC depolarization will be disrupted by retroactive control.

In the mammalian brain there are indications that different functional micro-circuits within the prefrontal cortex are activated depending on the utilization of the proactive or retroactive control^47^. Here we tested whether it is also the case when different types of training paradigms are combined in *Lymnaea*. We show that unlike appetitive training, aversive conditioning has no effect on the CGCs but the aversive conditioned stimulus triggers increased spiking activity in the PlB that is part of the defensive-withdrawal network^31^. In humans, procedural and declarative memories were thought to be encoded by distinct circuits^48^. However, it was shown that learning a declarative task followed by a procedural task could cause retroactive interference with the first memory, and vice versa^49^, suggesting that there is interaction between the two memory systems. In *Lymnaea*, the circuit mechanisms identified for encoding the appetitive and aversive memories are distinct, since they utilize different neural pathways and this is further supported by the lack of interference between the memories when the second training is performed at the non-lapse. However, any new learning during the lapse period, regardless of circuit mechanisms, results in retroactive interference. It is possible that there is some interaction between the memory systems during the acquisition phase of the new memory, but that the original memory is only vulnerable to interference when it is in a labile state. Since both memories act on the same output network (the feeding system) there could indeed be interactions during acquisition of the new memory, but not in their long-term storage mechanisms. Future experiments are needed to determine whether the memory circuits interact during the acquisition of new information.

Our findings allowed us to establish the time and circuit dependence of retroactive versus proactive interference during memory consolidation, paving the way for future work in our and other systems aimed at revealing their underlying molecular control mechanisms.

## Methods

### Animal maintenance

Snails (*Lymnaea stagnalis*) were kept in groups in large holding tanks containing Cu^2+^-free water at 20 °C on a 12:12 h light-dark regime. The animals were fed lettuce three times a week and a vegetable based fish food (Tetra-Phyll; TETRA Werke, Melle, Germany) twice a week. Animals were transferred to smaller holding tanks of 10 animals per tank, and food-deprived for 2 days prior to experiments. For all the experiments adult (3–4-months-old) snails were used.

### Behavioral training and testing procedures

Single-trial appetitive conditioning was performed by pairing either gamma-nonalactone (0.004%) or amyl-acetate (0.004%), the conditioned stimuli, with sucrose (0.33%), the unconditioned stimulus, using a previously well-described method^8,19,50^. Briefly, animals were placed individually in petri dishes containing 90 ml Cu^2+^-free water and allowed to acclimatize for 10 min before the training procedure started. Training started when 5 ml of the conditioned stimulus was added to the water and 30 s later 5 ml of the unconditioned stimulus was added to the dish. Animals were left in the solution containing both the conditioned and unconditioned stimulus for 2 min and then they were rinsed in Cu^2+^-free water before being returned to their holding tanks. Animals were tested for their conditioned response 24 h after training. Animals from trained and naïve groups were transferred from their holding tanks and placed individually in petri dishes containing 90 ml Cu^2+^-free water and allowed to acclimatize for 10 min. Testing began when 5 ml of water was added to the petri dish. The number of feeding cycles that the animal performed was counted over a 2 min period after the water was added to the dish. A single feeding cycle consisted of the visible sequence of movements of the mouth-parts consisting of the opening of the mouth and the protraction/retraction of the toothed radula followed by the mouth closing. Next, 5 ml of the conditioned stimulus was added to the dish, and the number of feeding cycles was counted during the subsequent 2 min. Conditioned responses were assessed by calculating a feeding ‘difference score’. The difference score was obtained by subtracting the number of feeding cycles observed during the 2 min after water application from the number of feeding cycles in the 2 min after conditioned stimulus application. A blinded procedure was used in the behavioral tests. The protocol for the single-trial aversive conditioning protocol was the same as the appetitive one described above, however the conditioned stimulus used was L-serine (0.011%) and the unconditioned stimulus quinine (0.075%). To investigate the timing of the lapses during memory consolidation, conditioning was performed as above but memory recall was tested at 10 min, 30 min, 1, 2, 3, 4 or 24 h after the pairing of gamma-nonalactone (0.004%) and sucrose (0.175%) (Supplementary Fig. 1) or L-serine (0.011%) and quinine (0.075%) (Supplementary Fig. 3) in separate groups of animals.

During dual conditioning experiments, animals first received gamma-nonalactone paired with sucrose as the first training, which was then followed by the second training using either amyl acetate paired with sucrose or L-serine paired with quinine. The second training was applied at either a lapse (2 h) or non-lapse point (1 h) of the first memory in different groups of animals. In Fig. 3e, f, animals received L-serine paired with quinine first followed by gamma-nonalactone paired with sucrose. Conditioned stimulus responses were obtained 24 h after the onset of the first training. The protocol for backwards conditioning of the unconditioned stimulus + conditioned stimulus was similar to the forward conditioning described above, however the unconditioned stimulus was applied first for 2 min followed by a brief rinse in Cu^2+^-free water after which the animal was placed in a new petri dish where they received the conditioned stimulus for 2 min. Testing after backwards conditioning was the same as the forwards conditioning described above. In pharmacological experiments, the protein synthesis blocker anisomycin was injected into the hemocoel of the animal via the foot. Animals were injected with 100 µl anisomycin (Sigma) in normal saline (0.1 mM final concentration) after training. Control animals were injected with normal saline alone after training and behavioral testing was performed as above.

Sensitization experiments were performed by applying tactile stimulation of the head (five times in 2 min) inducing whole-body withdrawal responses. Animals were then immediately placed in a chamber filled with Cu^2+^-free water, which held the animal at the surface of the water. *Lymnaea* often float on the water’s surface whilst feeding or searching for food, thus this design allowed us to record and analyze changes in their behavior in response to a stimulus in a behaviorally relevant manner^12,13^. Animals were allowed to acclimatize for 10 min in the chamber before a brief (600 ms duration) light off stimulus was presented. All animals were filmed at 33 frames/s. The total area of the head/foot complex was measured 1 s before the stimulus and at 3 time points after the stimulus (5, 10, and 20 s) in each animal using ImageJ software. The head/foot area was normalized to the before stimulus condition to compare changes induced by the stimulus. A reduced area therefore represented a withdrawal of the head/foot into the shell. Naïve animals underwent the same test but in the absence of the tactile stimulation of the head prior to testing.

### In vitro preparations and electrophysiological procedures

In vitro experiments were carried out using a lip-brain preparation or an isolated brain preparation. The lip-brain preparation used here is described in detail in Staras et al.^51^. Briefly, animals were dissected by making a dorsal incision to expose the brain, ensuring no damage was made to the lips/tentacles of the animal. All peripheral nerves were cut except the two medial lip nerves, two superior lip nerves and two tentacle nerves which convey sensory information from the lips/tentacles to the brain. The posterior region of the foot was removed. The lips were pinned in a Sylgard-coated dish and the brain prepared for electrophysiological recordings. The buccal, cerebral and pleural ganglia were de-sheathed using fine forceps and treated with a solid protease (Sigma-Aldrich) for 1 min to soften the inner-sheath. To test for differences in responses to the conditioned stimulus in naïve vs appetitive conditioned preparations, saline containing 0.004% gamma-nonalactone was applied to the lips for 2 min whilst recording intracellular activity from PlB to monitor changes in firing rates. PlB activity was analyzed by comparing firing rates in the 2 min preceding the conditioned stimulus application vs firing rates during the 2 min the conditioned stimulus was applied. We observed that unlike gamma-nonalactone, L-serine caused aberrant activity in central neurons. We therefore substituted L-serine application with its in vitro analogue, stimulation of the main chemosensory pathway, the medial lip nerve^52^. Stimulation of this nerve drives strong fictive feeding, similar to L-serine application in vivo (see Supplementary Fig. 4c). We used an isolated brain preparation for these experiments and stimulated the medial lip nerve using a glass suction electrode with biphasic pulses of 4 V with 0.5 ms duration at 1 Hz for 120 s. A fictive feeding difference score was calculated by recording activity in feeding motoneurons and counting the number of cycles which occurred in the 2 min period preceding medial lip nerve stimulation and subtracting this from the number of cycles in response to medial lip nerve stimulation. Fictive feeding responses were recorded in feeding motoneurons located in the buccal ganglia, such as B3. PlB firing was recorded for 2 min before and 2 min during the medial lip nerve stimulation to determine changes in firing rates due to conditioning. To measure changes in membrane properties of the CGCs, the two-electrode current clamp technique was used in isolated brain preparations^9,53^. The CGC’s membrane potential and action potential characteristics (action potential amplitude, half-width, and after-hyperpolarization amplitude) were determined over a 100 s period. All preparations were perfused with normal saline containing 50 mM NaCl, 1.6 mM KCl, 2 mM MgCl_2_, 3.5 mM CaCl_2_, 10 mM HEPES buffer in water. Intracellular recordings were made using sharp microelectrodes (5–20 MΩ) filled with 4 M potassium acetate. Axoclamp 2B (Axon Instruments, Molecular Devices) and NL 102 (Digitimer Ltd.) amplifiers were used and data acquired using a Micro 1401 mk II interface and analyzed using Spike 2 software (Cambridge Electronic Design, Cambridge, UK).

### Statistics and reproducibility

Data was analyzed using GraphPad Prism 6 (GraphPad Software) and expressed as violin and box and whisker plots. Heat plots were produced in MatLab (Mathworks). Each ‘*n*’ represents an individual animal in all behavioral experiments or an individual preparation for in vitro experiments. Animals were randomly assigned to either conditioned or naïve groups. The investigators were blinded to group allocation during the analysis of electrophysiological data. Each experiment was replicated at least twice. Normality was tested using the D’Agostino and Pearson omnibus normality test. Two-group statistical comparisons were performed using two-tailed unpaired t-test statistics or a Mann Whitney test. Data with more than two groups were first analyzed using a One-way ANOVA or a Kruskal–Wallis test. Subsequent comparisons were performed using either Bonferroni, Dunn’s or Dunnett’s post hoc test. The significance level was set at *p* < 0.05. No data were excluded from analysis.

### Reporting summary

Further information on research design is available in the Nature Research Reporting Summary linked to this article.

## Supplementary information

Supplementary Figures

Reporting Summary

## Data Availability

All data needed to evaluate the conclusions of the study are present in the paper and/or the Supplementary Material. Detailed numerical data are available on FigShare at https://sussex.figshare.com/s/16b0be65af996947a431 (10.25377/sussex.7825082)^54^. Additional data related to this paper may be requested from the authors.
