# Supplementary material for: Proactive and retroactive interference with associative memory consolidation in the snail Lymnaea is time and circuit dependent
Source: Commun Biol. 2019 Jun 26;2:242. doi: 10.1038/s42003-019-0470-y (PMC6595009; doi:10.1038/s42003-019-0470-y)
Supplement: Supplementary file 1 — Supplementary Figures [file 42003_2019_470_MOESM1_ESM.pdf]

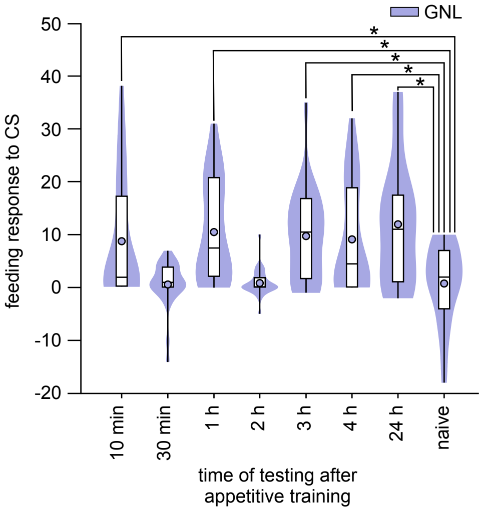


**Supplementary Figure 1. Lapses in memory recall following training with gamma-nonalactone + sucrose.** Lapses in memory recall occurred at 30 min and 2 h after single-trial appetitive conditioning. There was a significant increase in the response to the conditioned stimulus (CS), gamma-nonalactone (GNL), at all time points tested compared with naïve controls (n = 23) (10 min: n = 21, 1 h: n = 22, 3 h: n = 22, 4 h: n = 22, 24 h: n = 21) except at 30 min (n = 24) and 2 h (n = 24) (One-way ANOVA, $p < 0.001$ ($F_{(7,171)} = 6.99$), Bonferroni test: 10 min vs naïve $p < 0.05$, 4 h vs naïve $p < 0.05$, 1 h vs naïve $p < 0.01$, 3 h vs naïve $p < 0.01$, 24 h vs naïve $p < 0.001$, 30 min vs naïve $p > 0.05$ and 2 h vs naïve $p > 0.05$). Violin plots show density of data extending from minimum to maximum values. Internal boxplots show median and interquartile range (first and third quartile). Whiskers represent minimum to maximum values. Circles show the mean.

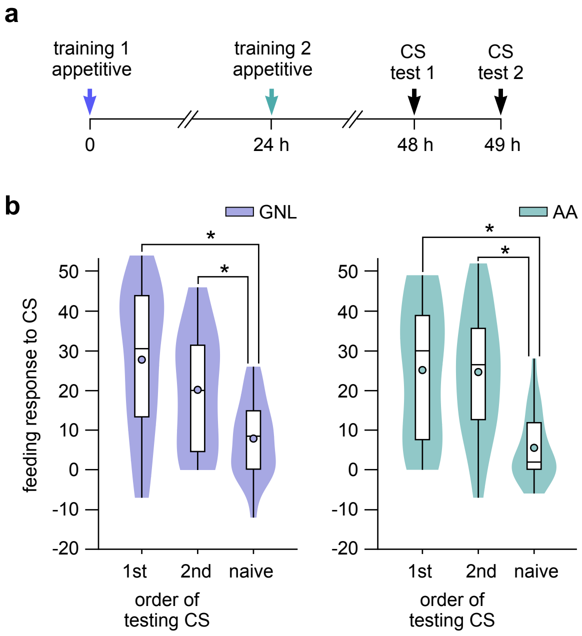


**Supplementary Figure 2. The same animal can store two appetitive memories.** (**a**) Time-line of experiment. Animals received the first training, gamma-nonalactone (GNL) + sucrose, followed 24 h later by the second training, amyl acetate (AA) + sucrose. The same animals were tested for their responses to both conditioned stimuli (CSs). (**b**) All animals showed a significantly greater response to gamma-nonalactone compared to naïve control animals, regardless of the order of testing (gamma-nonalactone 1$^{st}$: n = 24, gamma-nonalactone 2$^{nd:}$ n = 21, naïve: n = 22. One-way ANOVA, $p < 0.001$ ($F_{(2,64)}$ = 10.57), Bonferroni test: gamma-nonalactone 1$^{st}$ vs naïve $p < 0.001$, gamma-nonalactone 2$^{nd}$ vs naïve $p < 0.05$). When tested for their response to amyl acetate, the same animals also showed significantly greater responses compared with naïve (amyl acetate 1$^{st}$: n = 21, amyl acetate 2$^{nd}$: n = 24, naïve: n = 23. One-way ANOVA, $p<0.001$ ($F_{(2,65)}$ = 14.24), Bonferroni test: amyl acetate 1$^{st}$ vs naïve $p < 0.001$, amyl acetate 2$^{nd}$ vs naïve $p < 0.001$). Violin plots show density of data extending from minimum to maximum values. Internal boxplots show median and interquartile range (first and third quartile). Whiskers represent minimum to maximum values. Circles show the mean.

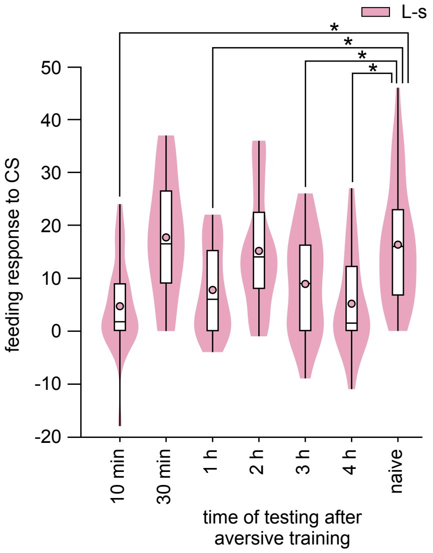


**Supplementary Figure 3. Lapses in memory recall following aversive conditioning.** Lapses in memory recall occurred at 30 min and 2 h after single-trial aversive conditioning. There was a significant decrease in the response to the conditioned stimulus (CS), L-serine (L-s), at all time points tested compared with naïve controls (n = 34) (10 min: n = 24, 1 h: n = 30, 3 h: n = 30, 4 h: n = 20) except at 30 min (n = 20) and 2 h (n = 29) (One-way ANOVA, $p < 0.001$ ($F_{(6,180)} = 7.37$), Bonferroni test: 10 min vs naïve $p < 0.001$, 1 h vs naïve $p < 0.01$, 3 h vs naïve $p < 0.05$, 4 h vs naïve $p < 0.001$, 30 min vs naïve $p > 0.05$ and 2 h vs naïve $p > 0.05$). Violin plots show density of data extending from minimum to maximum values. Internal boxplots show median and interquartile range (first and third quartile). Whiskers represent minimum to maximum values. Circles show the mean.

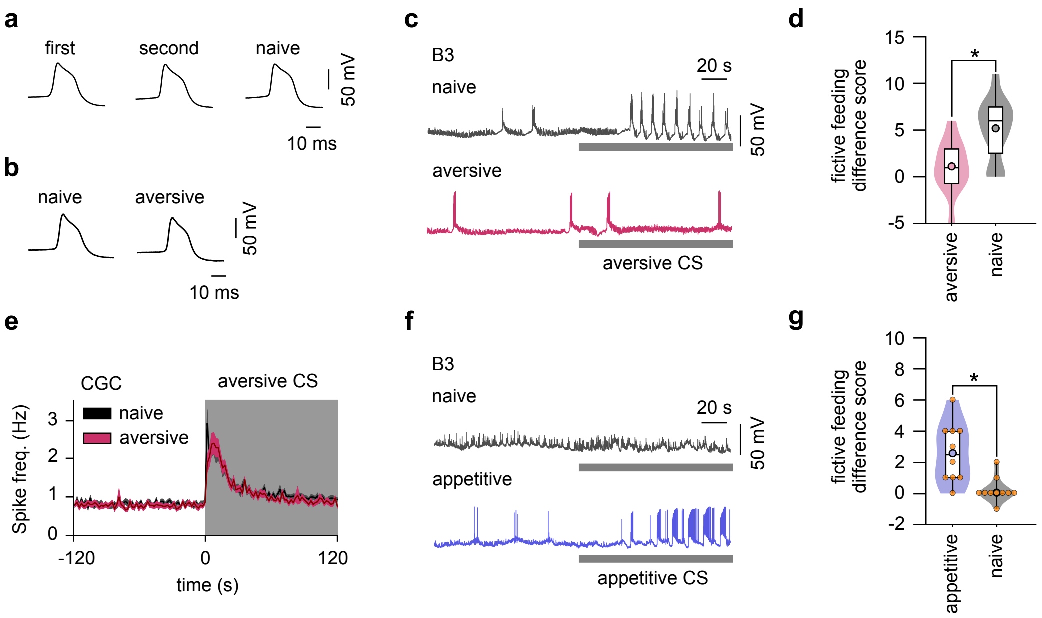


**Supplementary Figure 4. *In vitro* correlates of appetitive and aversive memories.** (**a**) Representative spikes from CGCs from animals which received the first training alone, second training alone or naïve animals. There were no statistical differences in spike characteristics between the two trained groups and naïve preparations. (Spike frequency (Hz) – first training alone: 0.93 ± 0.06, second training alone: 0.88 ± 0.04 naïve: 0.87 ± 0.05, One-way ANOVA, p = 0.71 ($F_{(2,35)}$ = 0.35). Spike amplitude (mV) – first training alone: 91.3 ± 0.7, second training alone: 93.8 ± 1, naïve: 93.4 ± 0.9, One-way ANOVA, p = 0.12 ($F_{(2,35)}$ = 2.29). After-hyperpolarization (mV) – first training alone: 13.1 ± 0.4, second training alone: 12.8 ± 0.4, naïve: 13.7 ± 0.4, One-way ANOVA, p = 0.30 ($F_{(2,35)}$ = 1.26). Spike half-width (ms) – first training alone: 15.8 ± 0.9, second training alone: 15.2 ± 0.6, naïve: 14.1 ± 0.9, One-way ANOVA, p = 0.32 ($F_{(2,35)}$ = 1.18). (**b**) Representative spikes from CGCs from animals that received aversive training or from naïve animals. There was no significant difference in CGC spike characteristics between aversively conditioned and naïve animals. (Spike frequency (Hz) – naïve: 0.77 ± 0.05, aversive: 0.83 ± 0.04, unpaired t-test, p = 0.36, t = 0.94, df = 22. Spike amplitude (mV) – naïve: 96.48 ± 0.63, aversive: 94.88 ± 0.56, unpaired t-test, p = 0.07, t = 1.89, df = 22. After-hyperpolarization (mV) – naïve: 12.38 ± 0.29, aversive: 12.72 ± 0.39, unpaired t-test, p = 0.50, t = 0.69, df = 22. Spike half-width (ms) - naïve: 13.13 ± 0.34, aversive: 12.88 ± 0.47, unpaired t-test, p = 0.68, t = 0.42, df = 22. (**c**) Representative traces of fictive feeding cycles monitored on a B3 motoneuron in response to the aversive conditioned stimulus (CS) in trained vs naïve preparations. (**d**) Statistical analysis of (c). The conditioned stimulus induced significantly fewer fictive feeding cycles in aversively conditioned preparations (n = 12) compared with naïve controls (n = 13, unpaired t-test, p = 0.003, t = 3.28, df = 23). (**e**) Line plot of CGC spike frequency in response to the aversive conditioned stimulus. There was no significant difference in CGC response to the conditioned stimulus between aversive conditioned (n = 21) and

naïve (n = 18) preparations (Mann Whitney test, p = 0.53, U = 166) and no significant difference in firing rates before the conditioned stimulus (aversive: 0.82 ± 0.027 Hz, naïve: 0.79 ± 0.045 Hz, unpaired t-test, p = 0.53, t = 0.64, df = 37). Data shows mean ± standard error of the mean. (**f**) Representative traces of fictive feeding cycles monitored on a B3 motoneuron in response to the appetitive conditioned stimulus in appetitive conditioned vs naïve preparations. (**g**) Statistical analysis of (f). The conditioned stimulus induced significantly more fictive feeding cycles in the appetitive conditioned preparations (n = 10) compared with naïve controls (n = 11, Mann Whitney test, p = 0.0006, U = 11). Violin plots show the density of the data points extending from the minimum to the maximum value. Internal boxplots show the median and interquartile (IQR; first and third quartile). Whiskers represent minimum to the maximum value. Blue and grey circles show the mean for appetitive and naïve respectively, orange circles show individual data points.

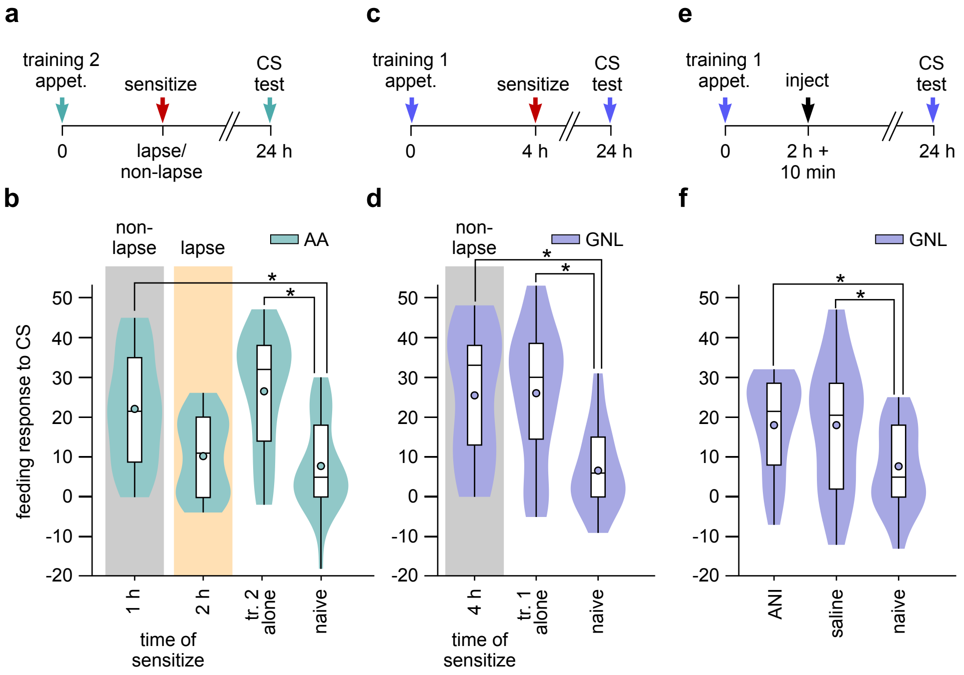


**Supplementary Figure 5. Sensitizing stimuli only disrupt memory when they are presented during a lapse.** (**a**) Time-line of appetitive conditioning followed by the sensitization behavioral paradigm. (**b**) Sensitizing stimulation is sufficient to block the expression of the second memory, amyl acetate (AA) + sucrose, when they are applied during the lapse (2 h) but not the non-lapse (1 h) point (second training alone: n = 27, 2 h: n = 23, 1 h: n = 24, naïve: n = 30. One-way ANOVA, $p < 0.001$ ($F_{(3,100)} = 14.03$), Bonferroni test: second training alone vs naïve $p < 0.001$, 1 h vs naïve $p < 0.001$, 2 h vs naïve $p > 0.05$). (**c**) Time-line of appetitive conditioning followed by sensitization behavioral paradigm (**d**) Presentation of the same sensitizing stimulation as in (b) does not affect the animal's memory for the first conditioned stimulus (CS), gamma-nonalactone (GNL), when presented at a non-lapse point, 4 h after training, compared with naïve controls (4 h: n = 19, first training alone: n = 29 naïve: n = 27. One-way ANOVA, $p < 0.001$ ($F_{(2,72)} = 14.04$), Bonferroni test: 4 h vs naïve $p < 0.001$, first training alone vs naïve $p < 0.001$). (**e**) Time-line of behavioral paradigm and pharmacological intervention (**f**) Anisomycin (ANI) injection does not affect the first memory when injected 2 h 10 min after training (ANI: n = 22, saline: n = 20, naïve: n = 19, One-way ANOVA, $p = 0.014$ ($F_{(2,58)} = 4.6$), Bonferroni test: ANI vs naïve $p < 0.05$, saline vs naïve $p < 0.05$, ANI vs saline $p > 0.05$). Violin plots show density of data extending from minimum to maximum values. Internal boxplots show median and interquartile range (first and third quartile). Whiskers represent minimum to maximum values. Circles show the mean.
